# Sentence comprehensionin Parkinson’s disease

**DOI:** 10.1590/S1980-57642008DN10400010

**Published:** 2007

**Authors:** Fernanda Prieto, Márcia Radanovic, Cristina Schmitt, Egberto Reis Barbosa, Letícia Lessa Mansur

**Affiliations:** 1 Speech Pathologist. Specialization in Neurolinguistics, Department of Physiotherapy, Speech Pathology and Occupational Therapy, University of Sao Paulo School of Medicine.; 2 MD, MSc, PhD, Department of Neurology, University of Sao Paulo School of Medicine.; 3 PhD, University of Michigan.; 4 MD, PhD, Department of Neurology, University of Sao Paulo School of Medicine.; 5 MsC, PhD, Associate Professor, Department of Physiotherapy, Speech Pathology and Occupational Therapy, University of Sao Paulo School of Medicine.

**Keywords:** Parkinson’s disease, comprehension, language tests, cognition, working memory, doença de Parkinson, compreensão, testes de linguagem, cognição, memória operacional

## Abstract

Parkinson’s disease (PD) patients with dementia have impairment of syntactic comprehension. Non-demented PD patients also experience difficulties in sentence comprehension and can be particularly impaired in the processing of grammatical characteristics of syntactically complex sentences.

**Objective:**

The aim of this study was to verify the performance of PD patients without dementia in a syntactic comprehension task compared with normal elderly.

**Methods:**

We studied oral sentence comprehension in fourteen patients with idiopathic PD together with fourteen controls matched for age and education, using the Token Test and Schmitt’s Syntactic Comprehension Test (developed in Brazilian Portuguese).

**Results:**

For the Token Test, there was no statistically significant difference between the PD group and the control group, whereas on the Syntactic Comprehension Test there was a slight statistically significant difference between the groups only for relatives in subject clauses (p=0.0407).

**Conclusions:**

PD patients differed from controls in the oral comprehension for relatives subject sentences alone. These results did not strictly reproduce those previously reported in the literature, and therefore point to the need for creating tests with diverse syntactic constructions in Portuguese able to produce consistent data regarding language behavior of Brazilian subjects with PD in comprehension tasks.

## Abstract (pt)

Pacientes com doença de Parkinson (DP) e demência apresentam prejuízo da compreensão sintática. Pacientes com DP sem demência também apresentam dificuldades na compreensão de sentenças e podem estar particularmente comprometidos na habilidade de processar as características gramaticais de sentenças sintaticamente complexas.

**Objetivo:**

O objetivo deste estudo foi verificar o desempenho de pacientes com DP sem demência em tarefas de compreensão sintática, comparados a idosos normais.

**Métodos:**

Foram estudados quatorze pacientes com DP idiopática e quatorze controles emparelhados por idade e escolaridade, usando o Teste Token e o Teste de Compreensão Sintática de Schmitt (desenvolvido em português do Brasil).

**Resultados:**

No Teste Token, não houve diferenças estatisticamente significantes entre os pacientes com DP e o grupo controle. No Teste de Compreensão Sintática, foi encontrada diferença estatisticamente significante entre os grupos apenas nas sentenças relativas de sujeito (p=0.0407).

**Conclusão:**

Os pacientes com DP diferenciam-se do grupo controle apenas na compreensão oral de sentenças relativas de sujeito. Tendo em vista estes resultados, que não reproduzem de forma estrita o que é descrito na literatura, torna-se evidente a necessidade da criação de testes de linguagem com diferentes construções sintáticas em português do Brasil, a fim de obtermos dados consistentes a respeito do comportamento de pacientes brasileiros com DP em tarefas de compreensão sintática.

Parkinson's disease (PD) is characterized by degenerative alterations in the dopaminergic neurons of the nigrostriatal pathways as well as in the noradrenergic, cholinergic and serotoninergic neurons in other cerebral regions. The degeneration in these pathways results in motor disorders that include resting tremor, bradykinesia, muscular rigidity, postural instability and gait disorders.^1^ As a rule, PD onset occurs between 40 and 70 years of age, most often at the beginning of the fifth decade. Until recently, any loss of cognitive function in PD was attributed to bradyphrenia (slow thinking). Currently, it is known that PD patients can exhibit loss of memory, attention, visuospatial skills and impairment to other cognitive domains. Furthermore, approximately thirty to forty per cent may develop “subcortical dementia”.^2^

PD patients with dementia have impaired syntactic comprehension which differs from that seen in Alzheimer’s disease (AD).^3,4^ However, until the 1980's language impairment was considered infrequent in PD without dementia. The first studies reporting deficits of sentence comprehension in PD patients without dementia were conducted by Lieberman et al.,^3,5^ showing that approximately half of the patients presented poor comprehension of complex syntactic constructions. The definition of complexity in these studies ranged from extension (number of propositions) to reversibility of thematic roles and modifications of canonical order (active constructions). Since then, numerous studies have demonstrated that non-demented PD patients also have difficulties in sentence comprehension and can be particularly impaired in the processing of grammatical characteristics of syntactically complex sentences.^6-11^

However, PD occurs in the elderly where subgroups with syntactical difficulties can be found, especially in producing left-branching sentences such as those relative in subject.^12-14^ At present, the exact nature of a possible functional disturbance explaining this impairment of comprehension remains unclear.

## Objective

The objective of this study was to verify the performance of PD patients without dementia in a syntactic comprehension task compared with normal elderly.

## Methods

### Subjects

Fourteen patients with idiopathic PD (stages 1 to 4 on Hoehn & Yahr scale, 1967 version)^15^ were evaluated at the Outpatient Unit for Movement Disorders from a tertiary university hospital. All patients presented scores within the normal range for the Brazilian population, adjusted for schooling, on the Mini Mental State Examination (MMSE).^16,17^ The time of disease varied from 1 to 26 years while age ranged from 52 to 75 years and schooling from 3 to 18 years. Portuguese was the native language for eight patients and Spanish for two, although these subjects spoke Portuguese as their main language. All patients were right-handed and were taking standard medication to control the disease during evaluation (levodopa, dopaminergic agonists, selegiline and anticholinergic drugs). Patients with history of other neurological and/ or psychiatric diseases, alcoholism, drug abuse or language acquisition disturbances were excluded. Ten normal subjects (four men), native Portuguese speakers, right-handed, aged between 46 and 72 years, with schooling between 4 and 11 years were evaluated as a control group. The control group was selected from the general population in accordance with the Mayo Older American Normative Studies (MOANS) criteria.^18^

### Material and procedures

The Token Test^19^ and the Syntactic Comprehension Test (Schmitt, not published) were used as instruments to evaluate sentence comprehension. The Syntactic Comprehension Test was fully developed by a linguist (CS) in Brazilian Portuguese.

The Token Test is highly sensitive to the alterations of language reception using immediate verbal memory as support. It promotes data concerning syntactic and semantic word levels, as the examinee needs to deal with a increase of message lenght and making semantic decodification of the words necessary to arrive at the meaning in the last section. The test is made up of five parts presenting progressive difficulty. In the first four parts, the commands are expressed in a form that uses elementary grammar and syntax, for example: Take *the small white circle and the small red rectangle*. In the fifth part, the test becomes more difficult from a linguistic point of view with the introduction of grammatical (prepositions, conjunctions and adverbs) and other more complex syntactic structures. In some cases, a small substitution changes the meaning of the command, for example: *Touch the blue circle with the red rectangle; and With the blue circle touch the red rectangle*.

The Syntactic Comprehension Test by Schmitt is composed of two batteries of 120 phrases each, based on the oral modality of Portuguese syntactic construction. The test includes active, passive, relative and interrogative sentences, with different concordance markers, as well as different mode, time and aspect markers. Comprehension Test 1 (CT1) contains 120 phrases composed of active and passive phrases; Comprehension Test 2 (CT2) contains 120 subjective phrases; Comprehension Test 3 (CT3) contains 40 interrogative phrases (see Appendix for details). The picture matching test method is used, i.e., the patient has to point to one of two pictures, corresponding to the phrase read by the examiner. The application of the tests was carried out in two sessions by a speech therapist specialized in Neurolinguistics.

All control subjects and patients signed a consent form prior to undergoing the evaluation. The study was approved by the Research Ethics Committee at the hospital where it was undertaken.

## Results

There were no differences related to gender (p=0.1428), age (p=0.2173) or schooling (p=0.3085) between the patient and control groups (demographic data on PD patients are shown in Table 1). On the Token Test, there was no statistically significant difference between the performance of the PD group and the control group (Table 2). Regarding the Syntactic Comprehension Test, there was a statistically significant difference between the groups only in those results for relatives in subject clauses (Table 3).

**Table 1:** Demographic data on PD individuals.

Subject	Gender	Age	Schooling (yrs)	Time of disease (yrs)	MMSE	H & Y Scale
1	M	52	4	10	28	1.5
2	M	57	3	11	27	2
3	F	58	3	26	29	4
4	M	58	16	12	29	2
5	M	58	16	15	25	2
6	F	63	4	11	25	2
7	M	65	18	3	28	2
8	M	66	3	6	28	2
9	M	68	8	1	30	1.5
10	F	75	11	15	28	1.5
11	M	71	5	21	29	1.5
12	M	66	4	4	28	1.5
13	M	5	8	7	29	1.5
14	M	55	8	10	26	1.5

H & Y: Hoehn & Yahr scale; MMSE: Mini-Mental State Examination.

**Table 2:** Performance of PD patients and controls on Token Test.

	Patients	Controls	p *
Part 1	10 (0)	10 (0)	0.9999
Part 2	9.5 (0.8)	9.8 (0.4)	0.7410
Part 3	9.8 (0.4)	9.9 (0.3)	0.4684
Part 4	8.4 (1)	8.9 (1.1)	0.3119
Part 5	15.8 (2.9)	15.2 (2.5)	0.5635

* Mann-Whitney Test.

**Table 3:** Performance of PD and control patients on Syntactic Comprehension Test.

Sentence structure	Patients	Controls	p *
Active	18.9 (0.9)	19.7 (0.7)	0.0522
Passive	19 (1.3)	19.1 (0.8)	0.9246
Active with topicalization	18.7 (1.4)	19.4 (0.8)	0.1466
Active with topicalization and reminder pronoun	19.2 (1.2)	19.3 (1)	0.8655
Passive with topicalization	18.8 (1.4)	19.5 (1)	0.1770
Subject relative	19.3 (0.7)	20 (0.3)	0.0407
Object relative	19.2 (1.4)	19.2 (0.9)	0.5318
Object relative with reminder pronoun	18.7 (1.2)	19.4 (0.7)	0.0937

* Mann-Whitney Test.

## Discussion

Sentence comprehension is a complex process that involves access to lexical items, syntactic construction, prosodic representation, thematic role attribution, propositional aspects and level of semantic discourse.^20^ PD patients exhibit impairment in memory, attention and language abilities such as deficits in verbal fluency, naming and comprehension of verbal information, as well as impairment in verbal and logical reasoning.^10^ This is because the disease affects the subcortical pathways involved in the activation of the prefrontal cortex, which in turn regulates speech production, syntactic skills and other aspects of cognition.

Based on the notion that sentence comprehension in the oral modality requires interpretation of the auditory sequential stimulus, with the need to “retake” the representation (from memory) of input elements that are missing or that need reinterpretation, there is considerable evidence that auditory comprehension normally involves immediate interpretation of the syntactic and semantic elements of the sentences being processed. Thus, the interruption of temporal processing or limited memory span, have significant effects on the ability of patients to understand the sentences.^21^

The working memory model is the basis for interpreting the difficulties in syntactic comprehension, a process that goes beyond the simple storing of information and includes the distribution of content to be processed, a role attributed to the central executive of the working memory system.^20^ An alternative view of working memory participation is that of Caplan and Waters, in which syntactic comprehension is based on widely-practiced differentiated storing processes making these independent and more efficient for on-line processing.^20,22^

Numerous studies have indicated the impairment of grammatically complex sentence comprehension in PD patients without dementia. Some authors have attributed this impairment to a primary deficit of allocating attentional resources by the central executive system,^7,23,24^ along with a major role of working memory deficit itself.^9,25,26^ This issue remains controversial, as some authors have failed to demonstrate a reduction in working memory span in PD patients,^27^ and the impairment in working memory span does not seem to be sufficient to affect sentence comprehension when only syntactic complexity is considered.^28-30^ On this point, Waters and Caplan have argued that many syntactic comprehension tests are based on off-line, post-interpretive processing, not reflecting the on-line processes of natural situations.^22^

The slower speed of information processing can also contribute to the previously described deficits in syntactic comprehension in PD.^23,24^ Some studies have suggested that PD patients can present significant delays in lexical activation.^31,32^ Finally, disorders of heuristic strategies for syntactic processing can be an additional factor in explaining comprehension deficits in PD.^3,5,8,11^

PD patients without dementia present diverse cognitive alterations, reflecting a disfunction of the striatal-thalamus-cortical pathways, including the dorso-lateral prefrontal pathways.^33^ An interesting advance in the understanding of the relationship among the various factors involved in the disturbances described above can be inferred from an fMRI study which demonstrated a decreased activation in the large scale network involved in syntactic processing, associated to a compensatory increase of activity in other cerebral regions in PD patients, compared to a control group.^34^ This pattern of compensatory activity, subject to interindividual variations and changes throughout the illness can explain the heterogeneity of the findings in the literature.

In this study, PD patients did not present difficulties in the Token Test or in the comprehension of active, passive and interrogative sentences presented in the standard syntactic structure of Brazilian Portuguese. Difficulties were found only for relatives in subject clauses. These results are intriguing, and possible explanations are:

a) the small number of patients in this study;
b) tests devised in Brazilian Portuguese are scarce and need to be applied in more extensive populations to detect their diagnostic sensitivity and provide a basis for their improvement;
c) this population, in fact, did not present any difficulties in sentence comprehension for simple and complex grammatical structure of Brazilian Portuguese.

The Token Test primarily relies on the addition of elements to be retained in order to accomplish the actions, being true even in the last part where the storing of information is highly dependent on language processing. On the other hand, the Schmitt test, upon adopting the grammatical patterns of the oral language, creates alert mechanisms, such as pronunciation emphasis or meaning redundancies, which our patients could have used as clues to achieve the correct answer. The exception would be the relatives in subject sentences where the elements fit in to the left, a construction which authors such as Kemper^13^ associated to short-term memory overload. Thus, it is necessary to create tests with diverse syntactic constructions in Portuguese, where patterns of stimulus presentation differ in the degree of information redundancy and pronunciation emphasis. Increasing the sample size is also advisable in order to achieve consistent data on the language behavior of Brazilian subjects with PD in comprehension tasks.

## APPENDIX

### Syntactic Comprehension Test – Examples of sentences for each syntactic construction

- actives: O camelo empurrou a girafa (The camel pushed the giraffe)
- actives + topicalization: A girafa o camelo empurrou (The giraffe the camel pushed)
- actives + topicalization + reminder pronoum: A girafa o camelo empurrou ela (The giraffe the camel pushed it)
- passives: A girafa foi empurrada pelo camelo (The giraffe was pushed by the camel)
- passives + topicalization: Pelo camelo a girafa foi empurrada (By the camel the giraffe was pushed)
- subject relative: Mostra o camelo que está empurrando a girafa (Show me the camel that is pushing the giraffe)
- object relative: Mostra a girafa que o camelo está empurrando (Show me the giraffe that the camel is pushing)
- object relative + reminder pronoum: Mostra a girafa que o camelo está empurrando ela (Show me the giraffe that the camel is pushing it)
- subject question: Que camelo está empurrando a girafa? (Which camel is pushing the giraffe?)
- object question: Que girafa o camelo está empurrando? (Which giraffe is the camel pushing?)
